# Punishment as a Means of Competition: Implications for Strong Reciprocity Theory

**DOI:** 10.1371/journal.pone.0120394

**Published:** 2015-03-26

**Authors:** Tünde Paál, Tamás Bereczkei

**Affiliations:** 1 Institute of Psychology, University of Pécs, Pécs, Hungary; 2 Institute of Psychology, University of Pécs, Pécs, Hungary; Hong Kong Baptist University, CHINA

## Abstract

Strong negative reciprocity, that is, sanctions imposed on norm violators at the punisher’s own expense, has powerful cooperation-enhancing effects in both real-life and experimental game situations. However, it is plausible that punishment may obtain alternative roles depending on social context and the personality characteristics of participants. We examined the occurrence of punishing behavior among 80 subjects in a strongly competitive Public Goods game setting. Despite the punishment condition, the amount of the contributions decreased steadily during the game. The amount of contributions had no significant effect on received and imposed punishments. The results indicate that certain social contexts (in this case, intensive competition) exert modifying effects on the role that punishment takes on. Subjects punished each other in order to achieve a higher rank and a financially better outcome. Punishment primarily functioned as a means of rivalry, instead of as a way of second-order cooperation, as strong reciprocity suggests. These results indicate the need for the possible modification of the social conditions of punishment mechanisms described by the strong reciprocity theory as an evolutionary explanation of human cooperation.

## Introduction and Theoretical Background

Among the most influential theories attempting to explain the evolution of human cooperation and altruism today we find kin selection, reciprocal altruism, reputation-based altruism, spatial reciprocity and the focus of the present study, the theory of strong reciprocity [1–22]. Strong reciprocity may take both positive and negative forms. A person is a strong positive reciprocator if he/she is willing to sacrifice resources to reward someone else who cooperates. In the case of strong negative reciprocity or social punishment, the individual who prefers cooperation is willing to sacrifice resources in order to punish or sanction those who free ride on others.

The research paradigm most commonly used for testing strong reciprocity theory is the Public Goods game, which is one of the most widely applicable models for so-called social dilemma situations [13, 21–23]. Such situations typically offer different decisional possibilities and behavioral alternatives in which individual and common interests are incompatible with each other. The behavior that would be beneficial for all is less profitable for the individual than the selfish alternative, yet it would severely harm the interests of the community if everyone followed self-regarding strategies [13, 24–26]. In each round, the players are provided with a certain amount of money, and they have to decide how much of it (if any) to contribute to a common account (i.e., the public good), and how much to keep in their own private account. In the traditional form of the game the amount contributed to the public good gradually declines over time, which can be explained by the fact that the players who cooperate do not do so at any cost; their cooperation is conditioned by the other group members’ behaviour [14, 27–28]. Cooperators, who would like to signal their dislike of the free riders’ strategy, in the absence of other options, retaliate by lowering their own contributions [14, 29].

If the players are given the chance to use targeted punishment against each other, the game unfolds differently [13–14, 27]. Obviously, when subjects are allowed to punish non-contributors, they do so at a cost to themselves. If punishing entails a considerable cost, it presumably serves as a sanction in the upholding of norms, and is imposed by the conditional cooperators. This is indeed what previous studies have found. According to concordant observations from these studies: sanctioning behavior follows the principles of strong negative reciprocity; the players who impose punishment even at their own expense are those who have an initially cooperative approach to the situation; sanctions are used against those participants who contribute nothing or only sub-standard amounts to the public good; and the existence of punishment opportunity results in a stable and high level of contributions, thus re-establishing cooperation [3–9, 11–15, 18, 30–32]. These corresponding observations show that the different manifestations of strong reciprocity are indeed effective devices for enhancing and maintaining cooperation. This is especially true for strong *negative* reciprocity: altruistic punishment is somewhat more stable and less vulnerable to free riding than altruistic rewarding [14].

From the perspective of the present study it is crucial to note that, depending on the experimental setup and the motivations and preferences of the participants, sanctions may have another function besides upholding norms. Accordingly, Ernst Fehr and Urs Fischbacher [31] differentiate between so-called *strategic* and *non-strategic* punishment. Non-strategic punishment is motivated by normative aggression in the service of upholding cooperation norms and enforcing fair behavior, and is based on the principle of strong negative reciprocity. This form of punishment serves cooperative functions as a secondary public good [15, 33]. When employing strategic punishment, however, the player aims to enhance his/her own profit regardless of whether the other participants are motivated by selfish or cooperative intentions. Fehr and Fischbacher [31] hypothesized that strategic punishment is not as strongly emotionally motivated as non-strategic punishment; the aim here is merely to obtain as much profit as possible by reducing the gains of the other participants. While Fehr and Fischbacher allowed for the existence of strategic punishment, they did not observe the evidence of its occurring, either in laboratory experiments or in real-life situations [31]. Comparing different versions of public goods experiments (partner condition vs. perfect stranger condition) they did not find any rigorous evidence that players are punished for strategic reasons.

In order to examine “pure” non-strategic punishment in the strong reciprocity paradigm, the authors set up an experimental situation in which the possibility of strategic punishment was removed [4, 6, 15]. While this experimental setting really allowed cooperation-enhancing processes to be examined through the Public Goods game, it provided certain simplifications compared to real-life circumstances. The various forms of punishment are necessarily intermingled in human social transactions where sanctions are involved, and strategic punishment cannot be entirely excluded. How can experimental settings for examining strategic punishment be provided? On the basis of the relevant literature, it is presumably the case that the punishment mechanism is especially sensitive to structural modifications to the experimental situation. It is highly relevant whether the punishment is costly or cost-free, whether it comes from other members of the group or from an external observer, and whether the group composition is stable or changes from round to round; group size and even the nationalities of the players are important modifying features as well [5, 30, 34].

One of the main conditions that strongly influence strategic punishment is the competitive style of the social situation. Intense competition presumably induces the players to enhance their gains by reducing the resources available to others. According to the results of field work, in circumstances where members of a work community compete with each other for the greatest benefit, the level of cooperation is lower than in communities where the mutually proficient strategy is cooperation [35–36].

One way of setting up a competitive situation is to modify a traditional social dilemma-type game according to the principles of the so-called *tournament theory* [37–39]. It has been soundly established both theoretically and empirically that when, instead of being rewarded on the basis of their *absolute* performance, the members of a group are rewarded on the basis of their *relative* performance, both the cooperative and the sanctioning behavior undergo substantial transformation [40]. In one of our previous studies we found that moving the emphasis from cooperation towards competition in a Public Goods game affects sanctioning behavior in a proportion of the players in a way that results in a form of punishing that is distinctly strategic [41]. Two of the most plausible means of achieving this are to change the payoff matrix and to modify the flow of information about the decisions of the other group members. Thus we altered two features of the standard Public Goods game: (1) After the final round of the game, the players received payoffs of fixed amounts, with the first-ranking player being substantially better off financially than the others; (2) In each round, players received information not only about the individual contributions and punishments in the given round, but also about the current financial standing of the others (see the section on Materials and Methods—Experimental situation). The first modification is likely to result in the players being unmotivated to raise their own profit by enhancing the net benefit of the group; instead, they will be motivated to obtain the highest profit in the group. The probable result of the second modification is that the targets of punishment will not be only the free riders but also all the players who are observed to have earned too much money. In the standard Public Goods game with costly punishment, participants are usually informed only about the individual contributions for the current round. Every player sees the others’ contributions, and if they regard the amounts as too low, they have the opportunity to impose sanctions. This threat induces the players to raise their contributions; otherwise, they may loose a substantial part of their profit.

However, if the participants are able to constantly monitor the financial standing of the other members—as occurred in our experiment—the situation may change fundamentally [42]. If they gain insight into the private accounts of the other players round by round, they are given an opportunity to punish those who have obtained an especially large profit. So they do not necessarily sanction those who contribute less to the public good, but rather those who are financially better off than themselves.

A possible consequence of the combination of these two conditions—a fixed amount payoff depending on final ranking, and insight into the current financial standing (thus the current ranking) of the other players—is an increased number and degree of strategic punishments. This is what we observe in the present study. The participants used punishment as a means of mutual rivalry, instead of as a device against free riding. The amounts of punishment was determined not by the contributions to the public good, but by the differences in the amounts of profit accumulated in the players’ private accounts. Thus, in this competitive situation punishment did not serve the function of inducing second-order cooperation but, instead, was applied as a means of rivalry for the highest profit.

### Hypotheses

#### Hypothesis 1

It is a general finding of multiple-round Public Goods games with costly punishment that the amounts contributed to the public good decline throughout the rounds without the opportunity for punishment, and have a tendency to increase in the rounds where there is the opportunity to impose sanctions [3, 7, 12–13]. This happens because strong reciprocators use the punishment option to sanction free riding, thereby indicating their disapproval and inducing free riders to contribute more to the public good. Also, those conditional cooperators who themselves tend not to punish, sensing that it is worthwhile to do so, begin to contribute larger amounts to common account again [3, 7, 12–13]. By contrast, we hypothesized that when competition within the group escalates, punishment looses its function of increasing the amounts of the individual contributions. Since in a tournament-type situation, mutual cooperation does not make financial sense, the players are interested exclusively in increasing their profit, and punishments are not directed at those who breach the cooperative norms.

#### Prediction 1

The average amounts of the individual contributions will not increase after the introduction of punishment; instead, they will remain at a generally constant level—or continue deteriorating—until the end of the game.

#### Hypothesis 2

Owing to the competitive characteristics of the experimental setting, we expected both strategic and non-strategic punishments to occur. We inferred the occurrence of strategic punishment if the amounts of received and imposed punishments had been influenced by the profits, but not by the contributions. If punishment functions as a form of secondary cooperation, we would expect those players to be punished whose contribution in the previous round deviated negatively from the mean contribution of the group. If, however, it is the amount of accumulated profit that elicits punishment, we could infer that a player imposes punishment in order to reduce another player’s profit and raise his/her own payoff. So, if the players punished the other members on the basis of the amounts of profit the other players had accumulated over the game rather than on the basis of these players’ contributions in the previous rounds, the punishment could be regarded as strategic.

#### Hypothesis 2.1

According to the principles of strong reciprocity theory, costly punishments are imposed by those players who are cooperative and thus contribute more to the common account, and it is imposed on those who contribute nothing or not enough to the public good [4, 8, 11–12]. Experimental findings have shown that the more a player’s contribution deviates negatively from the average contribution of the group, the more heavily he/she is punished. Correspondingly, the more a player’s contribution deviates positively from the average contribution of the group, the more punishment he/she imposes upon others. However, in a situation where punishment looses its cooperation-enhancing function, these expectations are unlikely to be met. We assumed instead that the amounts contributed would still have a certain effect on the levels of the punishments, but not to the extent of significance. We expected the occurrence of some non-strategic punishment based on observations found in relevant literature indicating that behavior considered to be unfair elicits strong negative emotions from some of the players, and this may constitute an element of the motivating forces behind sanctions [1, 9, 18]. Thus, we did not expect this motivation to be altogether absent here. However, given the characteristics of the experimental situation, we hypothesized that while some non-strategic punishment would be likely to occur, those who punish the most would not inevitably be the players who contribute to a greater extent to the public good, and the targets for the punishment would not necessarily be those players who contribute less or nothing to the public good.

#### Prediction 2.1

There will be no association between the punishments a player receives and imposes and the extent of deviation between his/her contributions and the average contributions of the other members of the group.

#### Hypothesis 2.2

In the “traditional” models of strong reciprocity the effect of the profit accumulated by the players on the punishment have not been examined. In the present study the players were given the opportunity to monitor the financial situation of the other group members in each round during the game. We expected that in this competitive situation strategic punishment would be more significant than non-strategic punishment. In other words, in the hope of the final payoff some players would systematically punish those group members who had the most money in their private accounts, irrespective of how cooperative they had been in the previous rounds. At the same time, the players who receive the highest amounts of punishment would be those who earned more money during the game relative to the others.

#### Prediction 2.2

The profit of the players relative to the average profit of the group will show a negative association with the punishment imposed by the players, and a positive association with the punishment received by the players.

#### Hypothesis 3

According to the findings of some earlier studies, one of the main motives behind antisocial punishment—where the sanctions are directed against the cooperators—is revenge [43–44]. In the standard Public Goods game, it is primarily the cooperators who punish the free riders. Some of the free riders do not tolerate this, and they retaliate. It has indeed been found that the amount of antisocial punishment increases in proportion to the received punishment. However, if punishment serves as a device for rivalry among players, it is improbable that it will be employed for retaliation and revenge. This is because revenge does not necessarily target those who have higher profits, but rather those who for some reason gave out punishments earlier. Consequently, revenge punishments do not increase the profit of the punisher relative to the other group members. Moreover, given that players did not receive information about the identity of their punishers, precisely targeted revenge would have been impossible in this situation.

#### Prediction 3

The punishment imposed by a player in a given round will be unrelated to the amount of punishment the same player received in the previous round.

## Results

### 1. The average amounts of the individual contributions over the games (Prediction 1)

**Fig 1:**
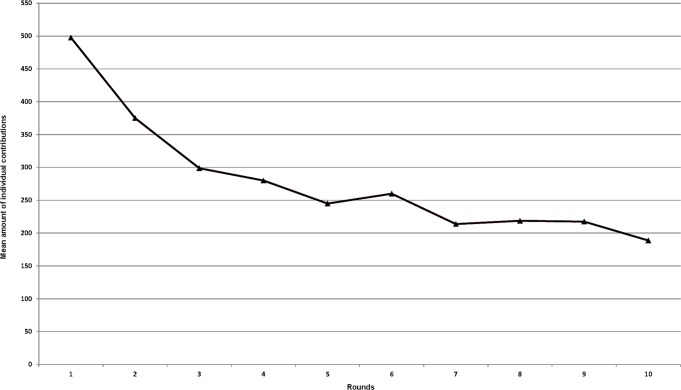
The average amounts of individual contributions to the public good throughout the game.

According to the results of the repeated measures ANOVA, the amount of the contributions deteriorated in a significant measure throughout the entire game: ANOVA: F _5.748, 454.088_ = 21.049, P *<*0. 001 (see Fig. 1). By separately analyzing the first half of the game (without punishment condition) and the second half (with punishment condition), we can see that the decline of the individual contributions is significant in the section from the first round to the fifth round: ANOVA: F _2.837, 224.128_ = 22.048, P < 0.001), and not significant in the section from the sixth round to the tenth round: ANOVA: F _3.631, 286.856_ = 1.993, P >0. 05). Our first prediction is thus supported. The average amount of the contributions decreased significantly throughout the game, and it did not increase after the introduction of the costly punishment condition. In other words, the decline of cooperation observed in the section without the punishment condition did not develop into an increasing tendency in the punishment section.

### 2. The relationship between the relative contribution of the players and the amount of punishment (Prediction 2.1)

In the light of the results of the linear regression analysis, the deviation in the contributions of individual players from the average contribution of the group does not explain the amount of the punishments received. We found no association with either the entire game or the individual rounds: the relative amount of a player’s contribution did not have a significant effect on the amount of punishment he/she received in the next round (see Table 1). The same is true for the imposed punishments: the relative contribution of the individual player in the previous round did not influence the amount of punishment imposed by that player in the subsequent round. Our expectations concerning the second prediction are thus confirmed: neither the amounts for the received nor the imposed punishments were influenced to a significant extent by the amount of the players’ contributions to the public good.

**Table 1:** Linear regressions for the relationship between the relative contributions of the indivual players and the amounts of received and imposed punishment.

Punishments	Relative contributions		
	*R* ^*2*^	*t*	Beta
Received punishment in the sixth round	.078	-.350	-.049
Received punishment in the seventh round	.070	-1.057	-.149
Received punishment in the eight round	.076	-.059	-.008
Received punishment in the ninth round	.039	-.520	-.070
Received punishment in the tenth round	.072	-1.265	-.158
Total amount of received punishment	.072	-.196	-.036
Imposed punishment in the sixth round	.031	.864	.124
Imposed punishment in the seventh round	.043	.902	.129
Imposed punishment in the eight round	.053	-.091	-.012
Imposed punishment in the ninth round	.077	-1.773	-.234
Imposed punishment in the tenth round	.071	-.307	-.038
Total amount of imposed punishment	.083	-.672	-.122

### 3. The relationship between the relative profit of the players and the amount of punishment (Prediction 2.2)

The third prediction was fully confirmed with regard to the imposed punishment, but only partly confirmed with regard to the received punishment. Table 2 demonstrates that the amount of the accumulated profit of the individual players had a strong influence on the amounts of punishment they imposed on others. By analyzing the individual rounds, we found that this effect first appears as a tendency in the eighth round, and then becomes significant in the ninth and tenth rounds: the less money a player had accumulated relative to the other group members during the previous rounds of the game, the higher the amount of punishment he/she imposed on the others. However, the deviation of individual profit from the sum of the others’ average profit did not influence the sum of the received punishments regarding the whole game. Only in the eighth round do we see a significant relationship, yet our data pointed in the expected direction for all other rounds, as well, although not at the level of significance.

**Table 2:** Linear regressions for the relationship between the relative profit of the indivual players and the amounts of received and imposed punishment.

Punishments	Relative profit		
	*R* ^*2*^	*t*	Beta
Received punishment in the sixth round	.078	1.754	.246
Received punishment in the seventh round	.070	1.037	.146
Received punishment in the eight round	.076	2.019	.270
Received punishment in the ninth round	.039	1.119	.151
Received punishment in the tenth round	.072	1.234	.154
Total amount of received punishment	.072	1.308	.239
Imposed punishment in the sixth round	.031	-.482	-.069
Imposed punishment in the seventh round	.043	-.712	-.102
Imposed punishment in the eight round	.053	-1.754	-.238
Imposed punishment in the ninth round	.077	-2.500	-.330
Imposed punishment in the tenth round	.071	-2.256	-.282
Total amount of imposed punishment	.083	-2.066	-.375

### 4. The relationship between the amount of received punishments and the amount of imposed punishments (Prediction 3)

The amount of punishment imposed by the individual players did not correlate significantly with the punishment received by the players in any of the rounds (Round 7: r = .124, P >. 05; Round 8: r = .15, P >. 05; Round 9: r = –.143, P >. 05; Round 10: r = .85, P > .05). Our fourth hypothesis was thus confirmed: the amounts of the received and imposed punishments do not correlate for individual players.

## Discussion

According to the strong reciprocity theory, cooperative tendencies should be positively selected for when sanctions are applied against those individuals who breach the norms of the community. Non-strategic punishment increases the level of cooperation within the group by forcing cheaters to raise their contributions to the community, and by providing the cooperators with the opportunity of repayment. Altruistic punishers regard the behavior of free riders as unfair and impose punishments motivated by their strong negative emotions, even if this punishment is costly to them [4, 6].

What happens, however, if the players apply strategic punishments instead of non-strategic ones? If instead of feeling unfairly treated, they are motivated by the will to win at any cost? In this case we may expect that sanctions will not function as second-order cooperation, that is to say, they will not enhance the level of the average contributions of the group. On the contrary, punishment becomes the device of intergroup competition, and the players will try to increase their profits by imposing sanctions on each other.

In order to examine this scenario, in the present study we designed a version of the Public Goods game where, in order to win, the participants needed to compete with each other. Not only were they provided with information about the contributions of the other players in each round, but also with the amount of total profit in each of the other players’ private accounts at the end of each round. We also included the condition that after the final round, players would receive payoffs of fixed amounts, with the player ranking first earning a substantially higher amount of money than the others.

Our first finding was that in this competitive context, the introduction of costly punishment did not reverse the decline of cooperation. This is in contrast to the usual result of the traditional experimental paradigm of strong reciprocity. The average net contribution decreased significantly throughout the game, and the costly punishment introduced—and effectively practiced—from the sixth round onwards did not change this tendency.

Our second finding concerned the relationship between the punishments and the contributions. In the standard model of strong reciprocity, the punishments received and imposed by a given player are a function of the deviation of his/her contribution from the average investment of the other members. However, we did not find such a relationship in our study: in neither round did the amount of the individual contributions predict either the amount of punishment received or punishment imposed by a particular player. From this result we can draw the conclusion that the sanctions were not intended to be a punishment of free riders, since in that case the players who were punished the most would have been those who contributed the least, and the players who imposed punishment the most would have been those who contributed to the public good more than the others.

Our third finding was that the profit gained by the players influenced the amount of the punishment they imposed on others. The players used sanctions if their profit deviated negatively from the others’ average profit, regardless of how much they or the others contributed to the public good. The results of the regression analyses suggest that non-strategic punishment, a characteristic of strong reciprocators, played a relatively smaller role in this competitive situation, while the strategic type of punishment played a much more determining role. Instead of imposing punishment in order to enhance cooperation, the players applied it as a device for intergroup competition.

The results related to the second pair of predictions reveal why costly punishment did not stop the deterioration of cooperation throughout the game. Strategic punishment dramatically reduces the individual contributions since it weakens the motivation to cooperate that would have been observed if the players only punished the defectors. If both defection and cooperation is sanctioned, free riders do not feel compelled to revert to cooperation, while cooperators do not feel encouraged to contribute to the public good.

Our results are in accordance with those of certain earlier studies. In some Public Goods game-type experiments, it has been found that a small but significant proportion of the punishment was imposed on those cooperators who earned above average during the earlier rounds of the game [30, 45]. A study carried out in India revealed that high-caste subjects had imposed severe sanctions out of spiteful motivations, irrespective of the other subjects’ decisions’ fairness or lack thereof [46]. Sanctioning cooperators may also serve as revenge, in the sense that the defectors retaliate for the punishment they received before from the cooperators themselves [44].

The question arises of whether the patterns of strategic punishment found in our study and their effects on cooperation may not be interpreted in the light of some other, already described punishment mechanisms. In some studies punishment is termed as strategic or *spiteful* if it is employed by someone who thinks that others threaten his/her advantage. This concurs with our results. However, in the situations described in these studies, strategic punishment occurred if its costs were low, and the sanctioning behavior itself did not take place in a competitive situation, but in the setting of a Public Goods game based on the standard payoff matrix [30, 46]. Thus, free riders punished only if it was possible to significantly reduce the other players’ profit at a small cost to themselves. On the other hand, they did not punish in those settings where it would have threatened their own profit. In sharp contrast—and in accordance with the principles of strong reciprocity—cooperative players placed sanctions on free riders even when this did not imply financial advantage for them, because the cost of the punishment was exactly the same as the punishment itself. In our study a different type of punishment mechanism was observable.

The case is similar for *antisocial* punishment, which is imposed on participants who contribute the same or more to the public good as the punisher, yet at the same time it is not necessarily beneficial for the punisher either [43–44, 47]. From the results of a cross-cultural study, Hermann, Thöni, and Gaechter [44] inferred that antisocial punishment may have several functions and, accordingly, several underlying motivations including a striving for dominance, competitiveness, retaliation, derogation of “do-gooders” etc. Our results differ from the model of antisocial punishment in three aspects. First, in our study the amounts of the contributions do not explain the amounts of received punishments; that is, the punishments were directed towards those players who had higher profits than the punisher, regardless of whether the punished players were cooperative or uncooperative. Second, sanctions employed in the service of competition were definitely financially beneficial for those who used them. Thirdly, we have found no evidence that punishment could have been used as retaliation for previous sanctions. Indeed, the results indicate the opposite relationship: the punishments actually imposed by the players were independent of the amount of punishment they had themselves received in the previous round (See Results 4.—Prediction 3).

As mentioned above, in the present experimental paradigm we introduced payoff conditions based on the tournament theory, thus creating a situation where competition and the assertion of self-interest play crucial roles. These circumstances are obviously not uncommon in everyday life. Whichever human group we consider, it is often the case that while the individuals are trying to live up to certain group-level expectations, they also compete intensely for the limited resources. It is also not uncommon that after the competition, these resources get monopolized by only one or a few persons.

Such conditions are likely to have occurred often in the evolutionary environment [48–49], which may have implications for the theory of strong reciprocity. If there is intensive competition for limited resources within a group, non-strategic, altruistic punishers may become outnumbered by the strategic, non-altruistic punishers. In such an environment the positive selection for cooperation and altruism is not likely to work. This might mean that certain conditions defined until now by the strong reciprocity theory need a more exact, stricter redefinition.

One of the central tenets of strong reciprocity theory is that second-order cooperation provides the foundation for the integration of a group. According to theoretical computations and computer simulations, even a small number of altruistic punishers may be sufficient to maintain cooperation if the norms of the group function effectively, punishment is not too costly, there is a low level of intergroup migration, and the size of the group is small. One of the focal points of the theory is that selection sustains cooperation only if frequent punishment and strong sanctions are practiced [3, 8, 13, 15]. Under these conditions, the survival of the group depends on the presence and activities of the altruistic punishers (or rewarders), who can invade a population of self-regarding types.

The most important condition for the spread of strong cooperators is the establishment of group institutions that suppress inter-individual differences. If the opportunities for selfish strategies are restrained, individuals will not be able to accumulate resources to enhance their survival and reproductive success. Reciprocal rules of food sharing or collective decision-making mechanisms in a group might be examples of such cultural institutions during the course of human evolution [5]. Cooperative norms reduce the behavioral variability within the group, thus restraining the effectiveness of individual selection and heightening the relative importance of intergroup selection.

The problematic point is that if the opportunity to punish is introduced, strategic punishment within the group cannot be effectively eliminated. If the conditions of cooperation include sanctions against those who break the norms, this rule becomes applicable to others as well; that is, to all of those who, in the opinion of the punisher, own too many resources. The norm of cooperation is a two-edged sword; you can punish the defectors, but you also may punish those who for some reason have a surplus relative to your stock of resources, especially in circumstances that provide chances to compete with others and constantly monitor and control the group members. The model of strong reciprocity undoubtedly resolves a fundamental problem: cooperation can be maintained in a group if its members systematically punish free riders. But this introduces a new problem: how is it possible to ensure that punishment is imposed solely on behalf of the group?

The norms of cooperation and egalitarianism and their related institutions do not solve this problem in themselves, since they advocate that there should be no substantial wealth and social differences within the group. However, on the one hand, since strategic punishers are always present in the groups where competition occurs, their behavior is likely to maintain phenotypic variability. On the other hand, it is also a way of mitigating in-group differences if the punisher regularly deprives of resources those people who, relative to the punisher, has a surplus of wealth, status, sexual partners, and so on. Therefore, either individual or group selection is taken into consideration, and it is hard to conceive that the altruistic punishers invade the population and gradually replace the self-interested individuals, as the strong reciprocity theory claims.

Admittedly, the low number of participants in the present study imposes a significant limit on very far-reaching conclusions. However, the results do allow for further consideration. We modeled a situation where there was an opportunity to assert self-interest. Accordingly, we initially observed intense competition among the group members, which resulted in a decrease in cooperation. Punishment did not change this trend; the free riding strategy did not disappear from the groups. This happened because punishment became in large part the means of competition, which was used to increase the individual’s profit relative to the others. Altruistic punishment, which would originally have served to alter the behaviors that harm the interests of the group, did not play a determining role here in increasing and sustaining cooperation.

Obviously, for altruistic punishment to be positively selected for, strategic punishment has to be reduced to a minimal level. Further research is necessary to clarify what this level is, what conditions and relationships between strategic and non-strategic punishment are adaptive in a certain environment, and what kinds of social contexts ensure the evolution of cooperation comprising the joint functioning of these two types of punishment.

## Materials and Methods

### Participants

Eighty undergraduate students of a Hungarian university (32 male, 48 female) aged 19 to 25 (Mean *±* SD = 22.0,0 ± 2.11) were recruited. Approximately half of them were volunteers, and the others took part in the study as a course requirement. Anonymity was ensured. All participants received an attendance fee.

### Experimental situation

The experimental situation was a computerized, modified version of the original Public Goods game, programmed and conducted with the help of the *z-*Tree 3.2 (Zurich Toolbox for Readymade Economic Experiments) software [50].

The participants played in groups of four; the composition of the groups remained stable throughout the games. Participation was anonymous. The subjects were coded according to the letters of the alphabet and were placed in separate rooms, and they did not receive any information regarding each others’ identities either during or after the experiment. Before the first round, they received written instructions and, where necessary, verbal information about the rules of the game.

The game consisted of 10 rounds. The players were informed of the number of the current round by on-screen messages. Each player had a private account, and each group a common account. At the beginning of each round, the participants received 1000 points. They then had to decide whether they would like to contribute some of these points to the common account, and if so, how many. The points that were not contributed to the common good remained on the players’ private accounts and accumulated during the game. The points contributed to the common account were added and doubled by the program. This duplicated amount was then divided equally by four, and added to each player’s private account, regardless of the amount contributed by the respective players. This stage of the game consisted of five rounds. In the course of each round, the participants were informed by screen messages about the following: the profit they had made in the given round; the aggregate amount in their private accounts (termed the total profit) given at the end of the round; the contributions of the other group members in the given round; and the total profit of the other group members given at the end of the round.

The possibility of monitoring the entire payoff earned by the others over the whole game was one of the crucial modifications that we made to the Public Goods game. As mentioned previously, this experimental setting encouraged players to apply strategic punishment against those who had an actual profit above the group average, irrespective of whether they were cooperators or defectors in the previous rounds.

At the beginning of the sixth round, the players were given some new instructions informing them that from this round onwards, they would be able to reduce the total profit accumulated in the other group members’ private accounts. They had no knowledge of this punishment possibility before the sixth round. (To avoid ethical concerns, the term “punishment” was never used; the word “reduction” was applied instead). The maximum amount of punishment a participant could impose on another player was 500 points. They could punish all three other players, but they were also free to avoid giving punishments altogether. Punishment was not random: since the identities of the players remained fixed throughout the game (Player A remained the same in each round) the participants could choose whom to punish. Punishment was also costly: 25% of the imposed amount was subtracted from the punisher’s private account. The participants were informed by screen messages about the total amount of punishment they had received in the given round, and about the total cost of the punishment they had imposed in that round. They did not receive information about the identities of their punishers.

Besides monitoring others’ accumulating profit, the other crucial modification to the original setting of the Public Goods game concerned the payoff matrix. While in the overwhelming majority of experiments, the subjects have received an amount of money corresponding to the number of points they gained during the game, here they were paid a fixed amount of money, according to their final rank at the end of the tenth round. The most successful player took home a substantially larger sum than the others. Thus the player placed first with the most points received 5000 HUF (ca 20 EUR), the player placed second received 3000 HUF, the third received 2000 HUF, and the fourth received 1000 HUF.

### Ethics statement

Since both participation and analysis of data were completely anonymous, we did not obtain a written or verbal informed consent from the participants. We followed this procedure in accordance with the guidelines laid down in the Hungarian Code of Professional Ethics of Psychologists, paragraph 6.5.: “It is not necessary to obtain informed consent from the participants if there are well-founded reasons to presume that the research is in no aspect overburdening or damaging for the participants (e.g. observation of routine educational processes and methods, anonymous questionnaires, field observations etc.).” [51]. The participants received written information about the rules of the experimental game. We also ensured them that they were free to terminate participation any time during the experiment, and in this case their data would be immediately deleted from the records. At the end of the experiment participants were verbally debriefed, and again ensured that should they wish so, their data would be immediately, in their presence, deleted from the records. None of the participants expressed a wish to terminate the session or to have their data deleted.

We did not seek the approval of any ethics committee on the grounds that the approval of an ethics committee is necessary only if the research requires the permission of the institution wherein it is to be conducted. In our case it was not necessary to obtain such a permission. However, subsequent to the research we contacted the Ethics Committee of the Hungarian Psychological Association, and we received confirmation that neither the approval of the Committee nor informed consent from the participants had been necessary in this case.
